# Supplementary material for: Transgenic expression of Arabidopsis ELONGATION FACTOR-TU RECEPTOR (AtEFR) gene in banana enhances resistance against Xanthomonas campestris pv. musacearum
Source: PLoS One. 2023 Sep 1;18(9):e0290884. doi: 10.1371/journal.pone.0290884 (PMC10473477; doi:10.1371/journal.pone.0290884)
Supplement: S1 Table — (DOCX) [file pone.0290884.s001.docx]

**Supplementary Table 1**. List of primers used for qRT-PCR

| **Target gene** | **Primer sequences** |
| --- | --- |
| *AtEFR* | TGCTTGGCCCTGAGAATAAA |
|  | GGTTTCACATTCCGCCATAAAG |
| *Ma PR1-like* | CCGTCTGCTGTGTTCTGTAA |
|  | TTGCCGATACGTACTCATACAC |
| *Ma PR2-like* | GAAGGAGATCGAGGCGTACATA |
|  | GGGCTGCTTGTTGGGATAAA |
| *Ma PR3-like* | CAGCCAGAGTCCCTTTACTTAG |
|  | TCCTCCGCAACTTCTACAATC |
| *Ma PR4-like* | GGAAATGGAGGGCTGGATTTAG |
|  | CCCAGTCAACAAACTCGTAGTC |
| *Ma PR5-like* | ATGTTACTCCGACGATGCTTAC |
|  | TCAGAGCAGGCAACAATACC |
| *Ma WRKY-22 like* | TGGAGCCTCGTCTGATATGT |
|  | CTGCACCTGTAGTAACCTCTTG |
| *Ma 25s* | ACATTGTCAGGTGGGGAGTT |
|  | CCTTTTGTTCCACACGAGATT |
