# Supplementary material for: Transgenic expression of Arabidopsis ELONGATION FACTOR-TU RECEPTOR (AtEFR) gene in banana enhances resistance against Xanthomonas campestris pv. musacearum
Source: PLoS One. 2023 Sep 1;18(9):e0290884. doi: 10.1371/journal.pone.0290884 (PMC10473477; doi:10.1371/journal.pone.0290884)
Supplement: S1 Raw images — (PDF) [file pone.0290884.s002.pdf]

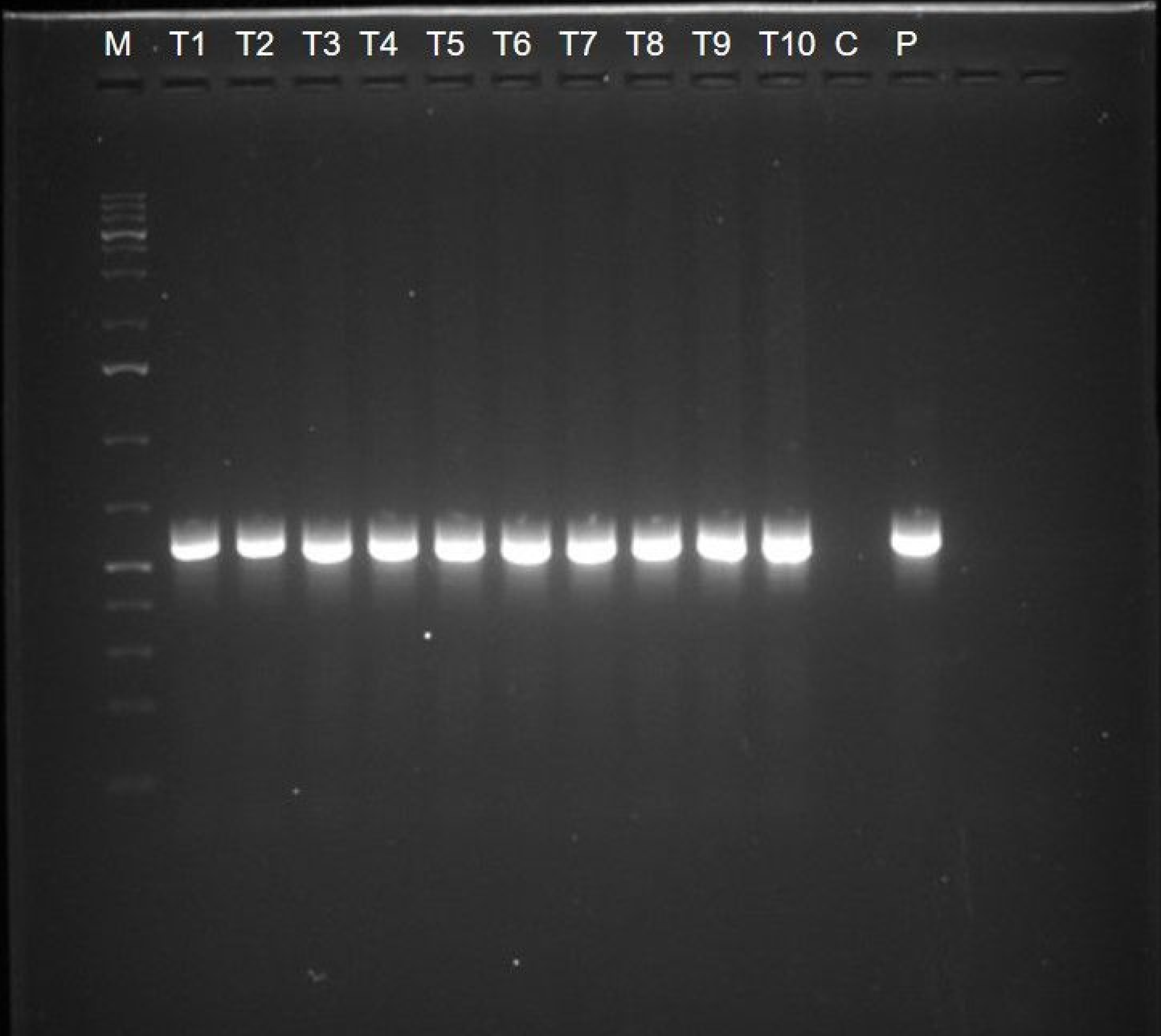

Fig 1 A. PCR products of selected AtEFR banana lines. The gel image was captured using SYNGENE imaging system.

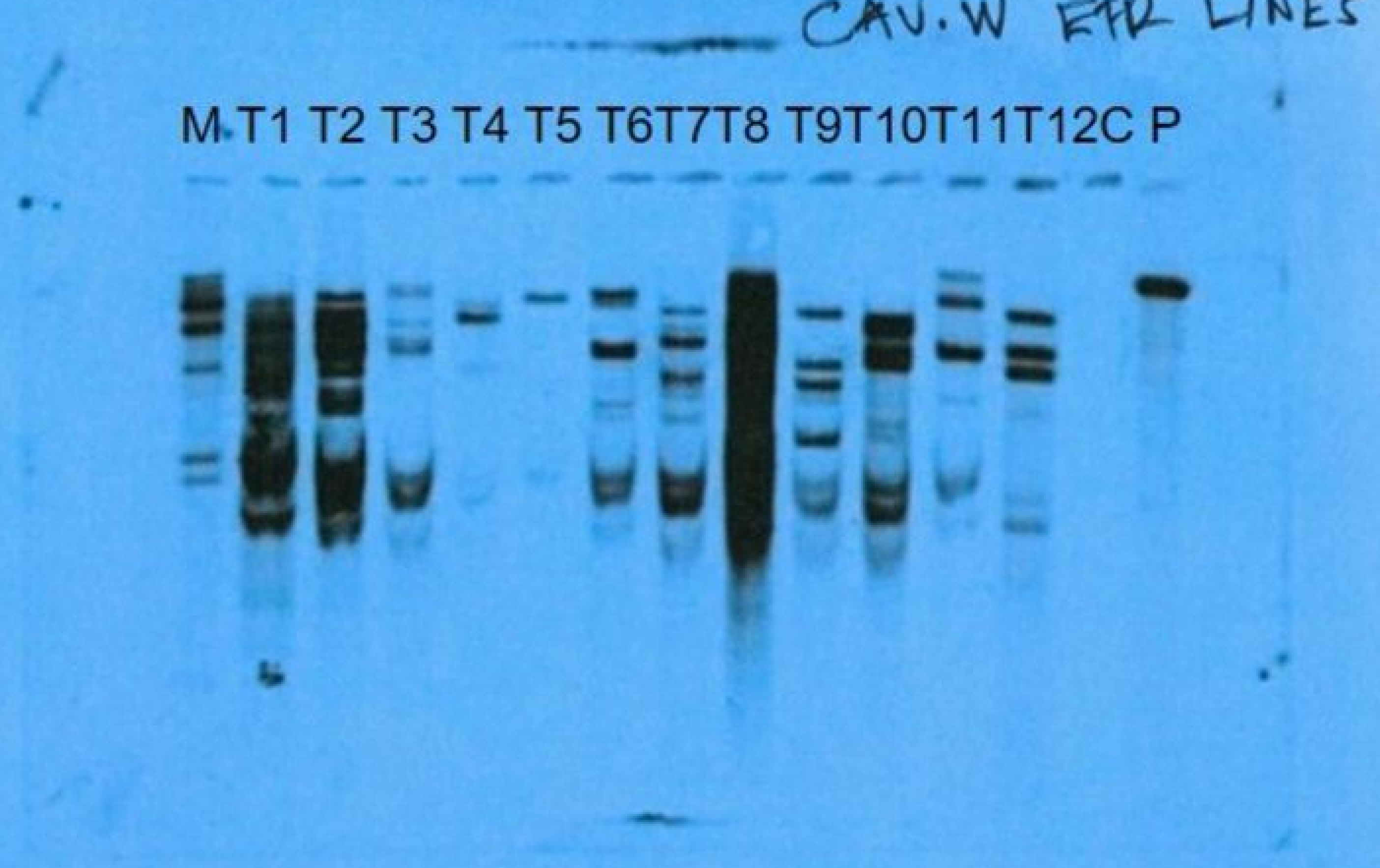

Fig 1B. Southern blot pattern of selected AtEFR banana lines. This X-ray film was developed in developer and fixer after 12 h exposure. The film was scanned using HP image scanner.

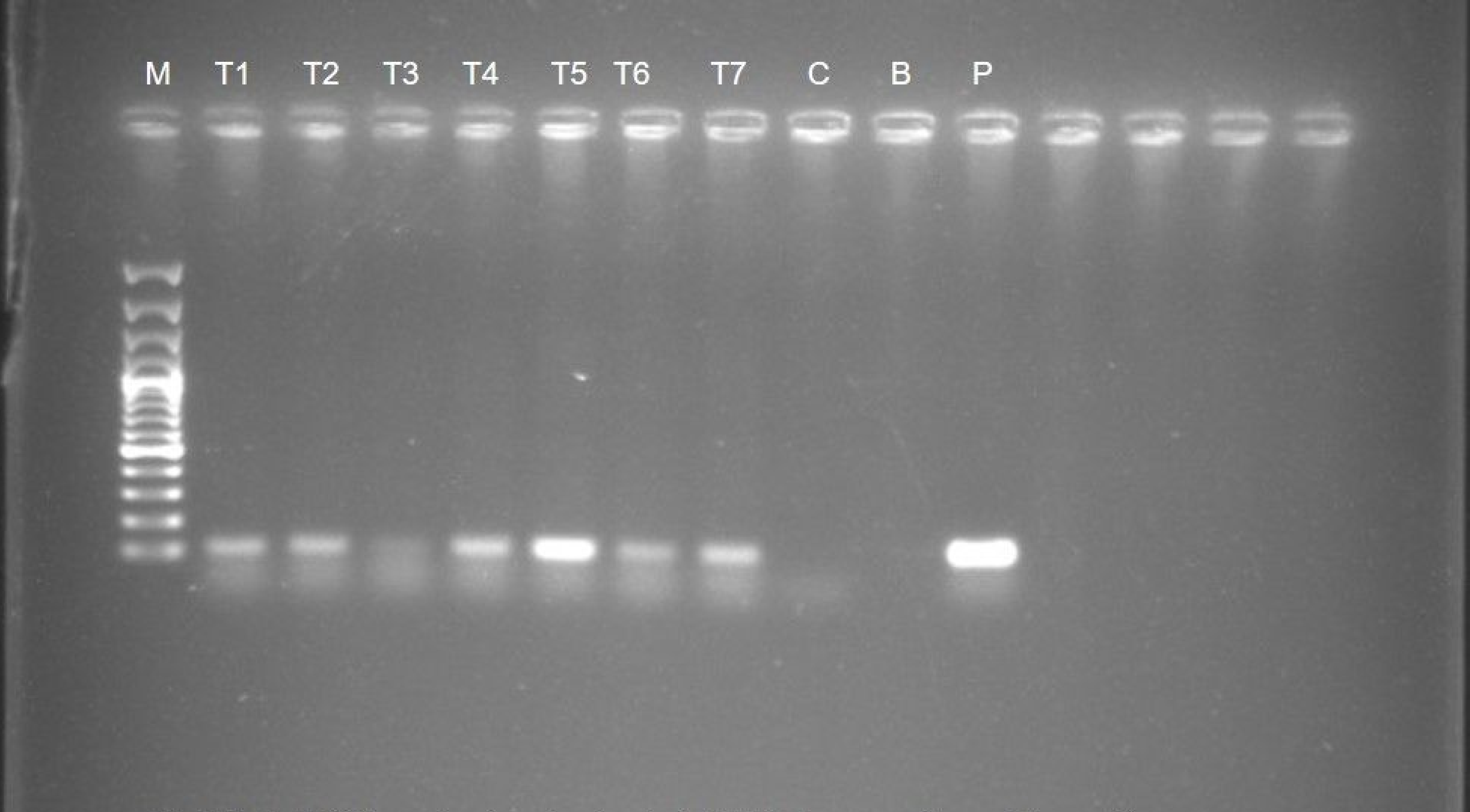

Fig 6A. RT-PCR products of selected AtEFR banana lines. The gel image was captured using SYNGENE imaging system.

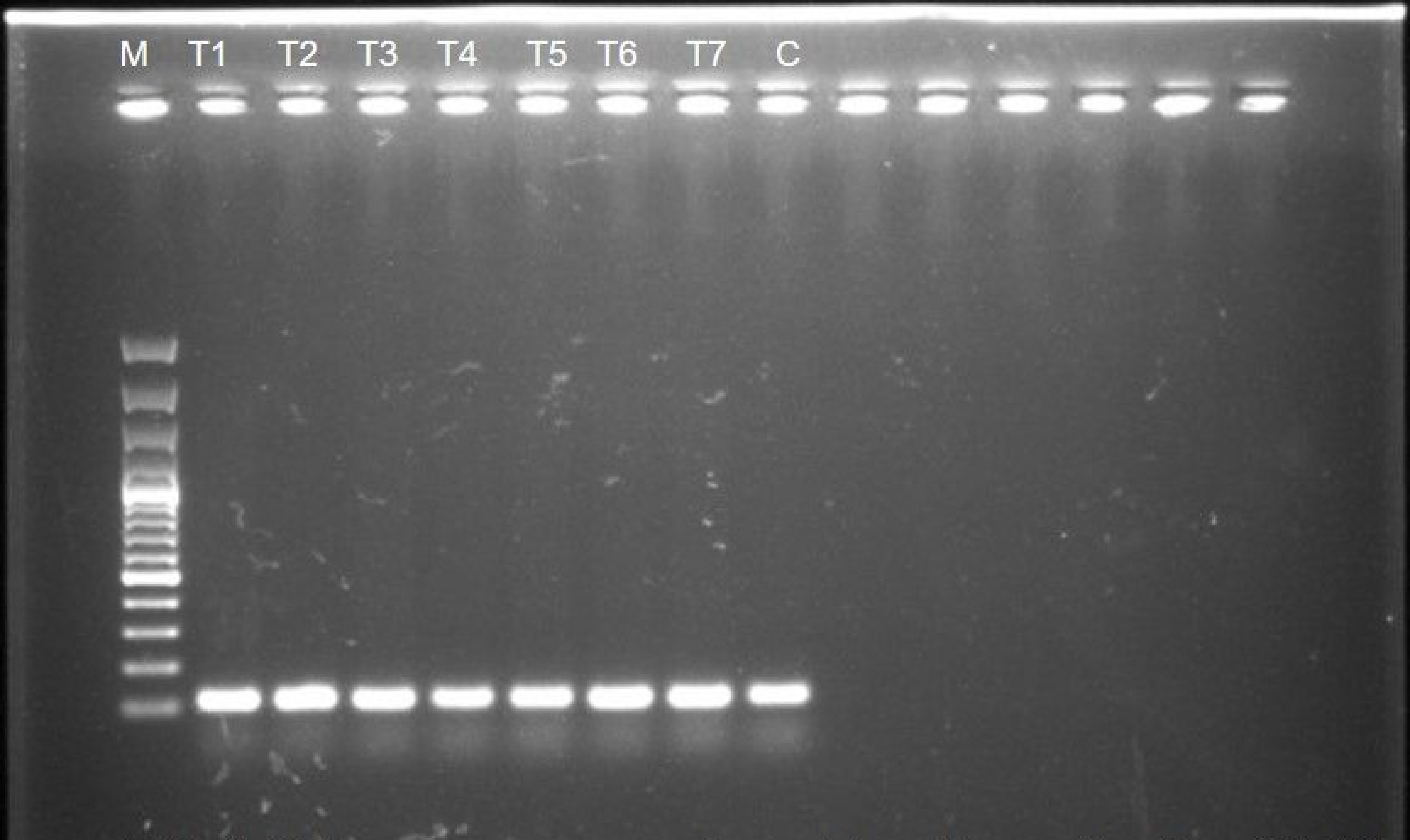

Fig 6B. RT-PCR products corresponding to *Musa 25s* gene in selected AtEFR banana lines. The gel image was captured using SYNGENE imaging system.
