# Supplementary material for: Transgenic expression of Arabidopsis ELONGATION FACTOR-TU RECEPTOR (AtEFR) gene in banana enhances resistance against Xanthomonas campestris pv. musacearum
Source: PLoS One. 2023 Sep 1;18(9):e0290884. doi: 10.1371/journal.pone.0290884 (PMC10473477; doi:10.1371/journal.pone.0290884)
Supplement: Attachment — Submitted filename: Response to reviewers.docx [file pone.0290884.s003.docx]

**Response to reviewer’s comments**

Dear Editor and Reviewers,

Thank you very much for considering our manuscript entitled ‘***Transgenic expression of Arabidopsis ELONGATION FACTOR-TU RECEPTOR (AtEFR) gene in banana enhances resistance to Xanthomonas campestris*’** for publication in your journal. We are also grateful for your constructive feedback on the manuscript. We have adopted your suggestions and corrected the manuscript with track changes. We now hope that the manuscript meets your expectation.

Our point-to-point response to the reviewer's comments is as follows:

**Reviewer #1**

1. It is suggested to expand on how the disease is spread in the field and to briefly outline the current forms of control.

**Response:** The information has been added in the introduction section.

2. The background of conventional breeding and sources of resistance seems underdeveloped. It is suggested to review the article by Nakato et al. (2019) 'Sources of resistance in Musa to Xanthomonas campestris pv. musacearum, the causal agent of banana Xanthomonas wilt'. Plant Pathology, 68, 49-59. Doi: 10.1111/ppa.12945. These authors report the evaluation of 72 banana accessions representative of Musa diversity for response to BXW by artificial inoculation. Some of the accessions reported in Nakato et al. (2019) e.g., M. acuminata subsp. zebrina could even be a good reference to contrast the effect of the atEFR gene with existing banana germplasm reported to be resistant/tolerant to BXW.

**Response:** This information is added to the introduction section. We have also explained how the knowledge from BXW-resistant *Musa balbisiana* can be transferred to BXW-susceptible banana cultivars.

3. Line 159: It is suggested to indicate the sublineage of the Xcm colony used in the assay.

**Response:** Xcm isolates from Uganda (sublineage 2) was used in this study. It has been added in the revised manuscript.

4. Line 179: Please check the expression. In planta bacterial population analysis?

**Response:** It has been revised.

5. Line 276: The southern indicates the number of insertions in the genome, and although it is correct that it could indicate the number of copies in the genome, it is very risky in the case of a single copy. It would be more correct to say that the number of copies of the transgene incorporated in the different events ranged from at least one to multiple (Fig. 1F). This is simply an observation and is at the discretion of the authors

**Response:** The sentence has been revised accordingly.

6. Line 360: Improve the expression of the abscissa legend in Figure 4 C.

**Response:** We have revised the figure legends (Figure 4C is now 6C).

7. Line 390: Please, check the expression. In planta, bacterial population analysis was performed.

**Response:** It has been revised.

8. Line 427: The EFR gene of A. thaliana (AtEFR) was previously overexpressed in several annual and some perennial species. From the point of view of gene efficacy, the assay shown in Table 1 seems more suitable for annual species, not so much in perennials. A more prolonged evaluation in time would seem more appropriate to know the effect of the AtEFR gene in banana cultivation. Moreover, the author’s opinion about their expectations of the durability of resistance in banana cultivation would be desirable.

**Response:** We have added a sentence in the discussion section to state that these transgenic banana will be further evaluated under field conditions for several generations to check the durability of disease resistance.

9. Line 435: Boschi et al. (2017) is quoted in two different ways: 17 and 36, please leave one alone. Check other quotes, e,g, 19 and 38.

**Response:** It has been corrected.

**Reviewer #2**

1. The scientific name of banana cultivar dwarf Cavendish is needed to be added, Musa spp. Cavendish group AAA.

**Response:** It has been added.

2. For experimental design, how many plantets per replicate?

**Response:** Three replicates were used for each transgenic line, this information has been included in the text.

3. In Fig 3, the The quality of the figures is not so good for publication. Please try to improve them.

**Response:** The figures have been revised (Fig 3 in now fig 4)

**Reviwer #3**

1. Include background information on the use of other transgenic approaches in banana for the control of Xcm.

**Response:** It has been added.

2. Why Figure legends are widespread along the manuscript and not necessarily in accordance with the text? Is really confused to follow the reading in this way.

**Response:** We have rearranged the figure legends to follow a logical sequence. Please note that the journal requires that we cite the figure legend immediately after its first mention.

3. The term “overexpressing AtEFR” is not correct and is repeated several times in the manuscript. This term is used to refer to gene expression in quantitative and relative terms from one condition to another. In this case the AtEFR gene is not overexpressed in the transgenic lines compared to the control plants (in fact in wt plants the AtEFR gene is not present and therefore not expressed).

**Response:** It has been revised.

4. The number and identity of transgenic lines used in the different activities performed varied without any justification or clarification of the selection criteria. Some activities were carried out with all the lines obtained and others with different subsets of selected lines that do not coincide with each other.

**Response:** The lines generated were subjected to PCR and southern blot analyses. The lines which were PCR positive were acclimatized and evaluated in the greenhouse. Lines for RT-PCR were selected based on their level of BXW resistance in the greenhouse. We selected lines that showed the highest BXW resistance and those that showed the least resistance for qRT-PCR analysis. For bacterial population assay, we selected the line which responded best in the bioassay (T5), the one which showed moderate response (T7), and the non-transgenic control. For the ROS assay and analysis of defense-related genes, we selected the line that had a single gene copy, showed the best response in the glasshouse and qRT-PCR (T5).

5. Additional information should be included in resistance assays descriptions: controls used (non-transgenic plants, non-inoculated plants), experimental design (randomized complete design, block design, etc).

**Response:** We have added the details.

6. Why AtEFR expression was assessed through both RT-PCR and RT-qPCR?

**Response:** RT-PCR was performed to check expression, but further quantitative expression was assessed through qRT-PCR.

7. Additional information for RT-qPCR analysis is required (primers, cycling conditions, etc).

**Response:** We have added the additional information. Primer details can be found in Table S1, which have been cited in the relevant sections.

8. Results of the southern blot assay are confused and not clearly presented. It is striking that only one line showed a single copy of the transgene (T5). The other lines showed multiple copies which is not a desirable trait. This fact should be made explicit in the text. Further characterization of these lines is also not justified. A more suitable way for copy number determination should be performed to confirm these results.

**Response:** It is indeed true that multiple copy is not a desirable trait. Also, Southern blot is not the most accurate approach for determining copy number. However, it is noteworthy that this is a proof of concept study mainly to determine if *AtEFR* can function in banana and if it can enhance resistance to EFR. The Southern blot was performed purposely to confirm gene integration and provide some rough information on the copy number. We will further evaluate these lines under field conditions and, if the resistance is sustained, conduct new transformation experiments using prefered banana cultivar in East Africa for product development. At this stage, we will conduct a more elaborate copy number analysis, incorporating other methods, especially because regulators do not prefer lines with multiple copy numbers.

9. The relative expression of AtEFR gene among transgenic events is part of their molecular verification but is presented separately after the resistance and the ROS assay.

**Response:** The verification of transgenic events was performed using PCR and Southern and then evaluated for disease resistance. We further checked gene expression in the selective transgenic events with varied resistance levels to check the correlation between expression and disease resistance. Variations in transcript levels were observed between the various transgenic events tested. For example, T5 event showed the highest level of AtEFR gene expression, whereas the lowest gene expression was observed in T3 event.

10. Figures must be organized logically and referenced in an orderly fashion in the text. Some figures cover more than one essay and are referred to in different sections of the manuscript, which makes it difficult to follow. - In the case of Figure 1, I suggest separating the E/F panels referring to molecular verification of the lines. Figure 3 should also be split in two. One part of the figure refers to the results of the resistance assays, showing the symptoms observed for one of the transgenic lines categorized as PR (T7) compared to the control. The other part of the figure shows the results of the ROS assay performed for the transgenic line T5. It is not understandable why both are shown in the same figure when they refer to different assays and transgenic lines.

**Response:** The figures have been re-arranged to ensure a logical flow.

11. Plant growth was analyzed for different transgenic lines but not for T5, the only one that carries the AtEFR gene in a single copy. This should be performed to complete the characterization of this line.

**Response:** Plant growth data was taken of eight randomly selected events prior to disease assay to check if expression of *AtEFR* has any negative impact on plant morphology. We did not observe any significant difference in the growth parameters of the transgenic events tested compared to the control non-transgenic plants. We felt that the eight events aree enough sample size to conclude that expression of AtEFR has no effect on plant growth. Therefore, we did not evaluated additional events for plant growth analysis. Anyways data will be collected for growth and agronomic parameters during confined field trials of the promising events.

12. Several references are repeated (Lu et al., Lacombe et al., Boschi et al., Piazza et al, Mitre et al., etc). Revise all.

**Response:** It has been corrected.

13. Lane 48: “BB genome”, “B genome”, explain or modify.

**Response:** BB is genomic group and B is the agenome.

14. Lane 163: “bacterial culture”. You mean “bacterial inoculum” or “bacterial suspension”?

**Response:** We meant bacterial suspension. This has been revised.

15. Lanes 163-164: remove “After inoculation” (is repeated at the end of the sentence)

**Response:** Done

16. Lane 183: Specify if the leaf-mid rib sections (1 cm) included the inoculation point or not.

**Response:** Yes, the mid-rib sections of the leaves were inoculated with the bacterial suspension.

17. Lane 213: “section 4.6”??

**Response:** It has been corrected.

18. Lanes 238-239: “between various transgenic events”…and control non-transgenic plants?

**Response:** It has been corrected.
